# Systematic review of pharmacological treatment options for orthostatic tremor in prospective patient cohorts and randomized controlled trials

**DOI:** 10.1007/s10072-025-08129-3

**Published:** 2025-03-29

**Authors:** Lena Sauthoff, Maider Iza Achutegui, Lan Ye, Clara Niesmann, Florian Wegner, Matthias Höllerhage, Martin Klietz

**Affiliations:** 1 Department of Neurology, Hannover Medical School, Carl-Neuberg-Straße 1, 30625 Hannover, Germany; 2 Department of Neurology, Vall d´Hebron Hospital, Paseo de la Vall d’Hebron, 119-129, Barcelona, 08035 Spain

**Keywords:** Orthostatic tremor, Shaky legs syndrome, Pharmacological treatment, Prospective trials

## Abstract

**Introduction:**

Orthostatic tremor is an infrequent movement disorder characterized by a high-frequency tremor manifesting primarily in the standing position. This condition can lead to relevant restrictions of mobility in everyday life and adversely affect the quality of life. The etiology has not been conclusively clarified. To date, there are few therapy studies of sufficient quality.

**Aim:**

The aim of the present literature analysis is to systematically evaluate the existing evidence of pharmacological therapies for orthostatic tremor.

**Materials and methods:**

This study searched for publications via PubMed and Google Scholar using the terms “orthostatic tremor” AND “therapy” and “shaky legs syndrome” AND “therapy”. The main inclusion criteria were a subject number ≥ 5, pharmacological treatment approaches and the presence of a prospective experimental design.

**Results:**

The evaluation of the results indicated the most positive evidence for therapy with gabapentin. Additionally, other drugs such as perampanel and levodopa also showed positive outcomes regarding specific endpoints. In contrast, there is a lack of evidence supporting the efficacy of levetiracetam and botulinum toxin in the context of primary orthostatic tremor.

**Discussion and conclusion:**

According to current evidence, gabapentin is the drug with the most robust data. However, further studies are needed to support the evidence for different pharmacological therapeutic approaches for orthostatic tremor. Future investigations should emphasize larger sample sizes, placebo-controlled, double-blinded methodologies and a longer follow-up to be able to make more precise recommendations with greater generalizability.

## Introduction

Primary orthostatic tremor is a rare neurological disorder with a still unclear incidence [1]. Those affected by primary orthostatic tremor suffer from a tremor with a frequency of 13–18 Hz that occurs exclusively when standing, leading to relevant restrictions in mobility, including an increased propensity to fall and an unsteady gait [2]. The symptoms may result in anxious and avoidant behaviors. Notably, patients are typically tremor-free while in sitting or lying position. The diagnosis of primary orthostatic tremor is established through the presence of a triad: unsteadiness in stance and possibly unsteady gait, otherwise unremarkable findings in the neurological examination and the typical tremor frequency in electrophysiological measurements [3]. The etiology of the disorder is classified as idiopathic, with insufficient evidence to identify specific triggering factors. However, the brainstem is suspected to be involved in its pathophysiology [4]. Some previous studies have examined the potential association between orthostatic tremor and the presynaptic nigrostriatal pathway, as well as its connection to the serotonergic system, yielding negative results [5]. Due to the limited understanding of the etiology, a causal therapeutic approach and prevention are currently not available. The therapeutic aim is merely to alleviate the symptoms, often through long-term medication. Current pharmacological approaches are based on measures that have already proven effective in other neurological diseases, although the mechanisms underlying their effectiveness in primary orthostatic tremor remain unclear. The drug management has so far been based on few studies with a very small number of patients.

The aim of this systematic review is to present the current evidence regarding pharmacological therapies for orthostatic tremor, thereby serving as a decision-making aid for treating neurologists and stimulate new research for the treatment of orthostatic tremor.

## Materials and methods

**Fig. 1:**
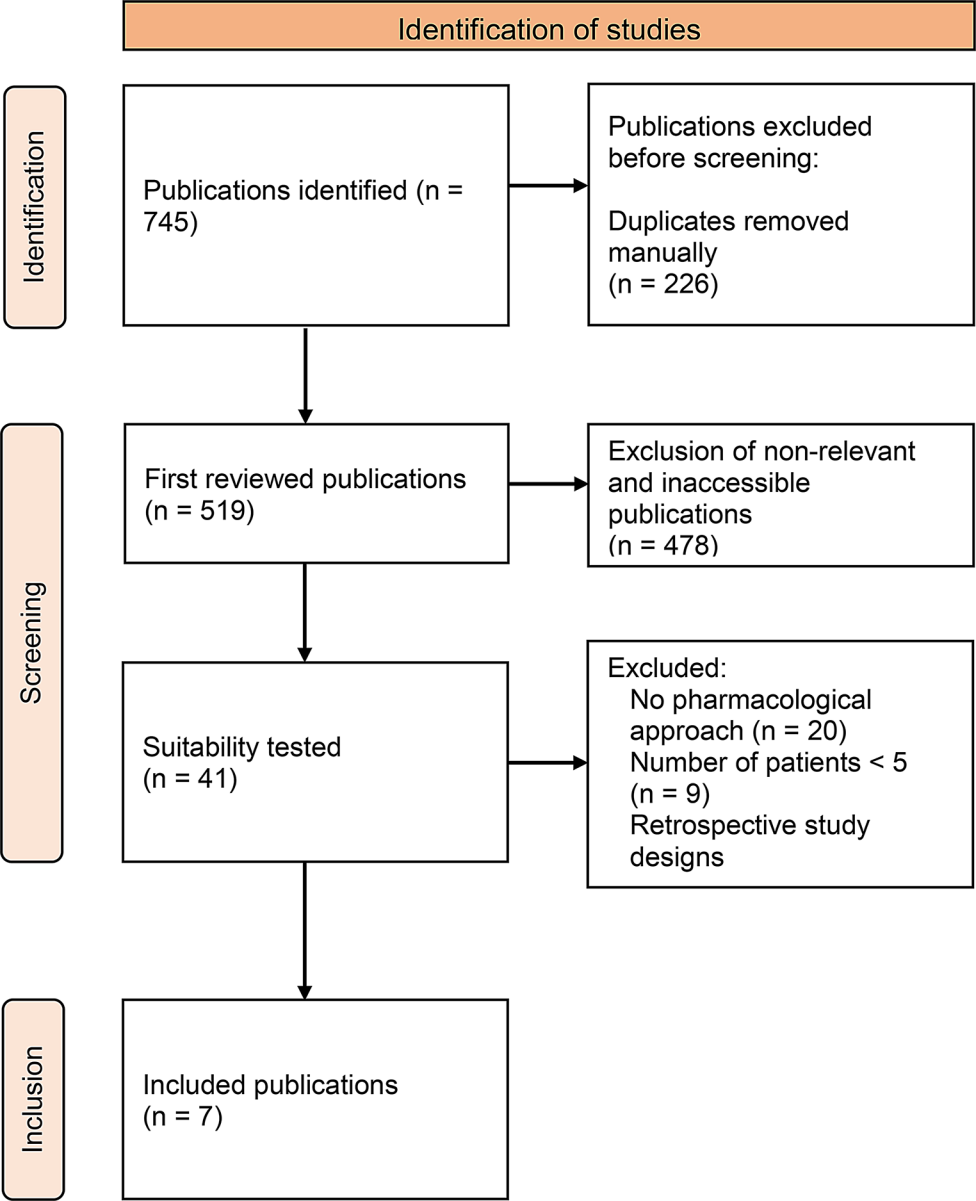
PRISMA flow chart

For the preparation of a systematic literature analysis, a digital search was conducted for suitable prospective studies. The databases utilized for this literature search included the meta-database PubMed and the search engine Google Scholar. The search terms used were “orthostatic tremor”; “orthostatic tremor” AND “therapy”; “shaky leg syndrome” and “shaky leg syndrome” AND “therapy”. Only studies in English language were included. The search revealed numerous overlaps, as illustrated in Fig. 1. The inclusion and exclusion criteria were defined prior to initiating the literature search as can be seen in Fig. 1. Studies were deemed eligible for inclusion if they had a prospective design, included at least 5 patients per treatment arm, and reported disease-relevant outcomes. Conversely, studies that did not report outcomes, recruited fewer than 5 patients per treatment group, comprised case reports or involved animal studies were excluded from the analysis. Invasive non-pharmacological treatment options were excluded in this review, a recent review of these was performed by Boogers et al. [6]. The systematic literature analysis was performed 12/2023. However, before submission of the manuscript no additional publications matching the inclusion criteria could be identified.

**Fig. 2:**
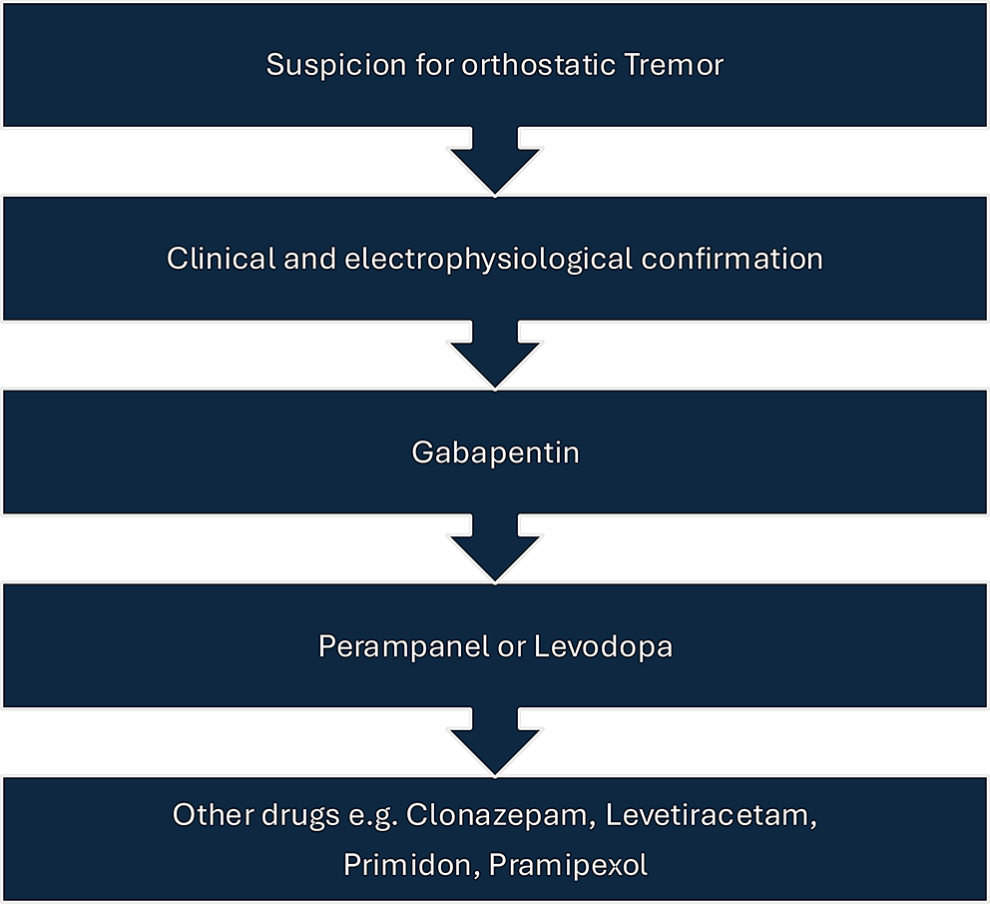
Flowchart: Recommended diagnostic and treatment approach for orthostatic tremor

## Results

Seven studies were identified that met the above-mentioned criteria and were considered suitable for analysis; three studies examined the efficacy of gabapentin, while one study each focused on the efficacy of botulinum toxin, levetiracetam, levodopa and perampanel (Table 1).

**Table 1:** Overview of the selected studies

Medication	Botulinumtoxin	Gabapentin	Levetiracetam	Levodopa	Perampanel
Number of studies	1	3	1	1	1
Authors	Bertram et al. (2012)	1) Rodrigues et al. (2006) 2) Evidente et al. (1998) 3) Rodrigues et al. (2005)	Hellriegel et al. (2011)	Wills et al. (1998)	Gironell and Marín-Lahoz (2019)
Number of included patients	8 (dropout: 1)	6 (dropout: 0) 7 (dropout: 0) 6 (dropout: 0)	12 (dropout: 1)	8 (dropout: 3)	20 (dropout: 8)
Type of study	Randomized, double-blind, placebo-controlled, crossover	1) Double-blind, placebo-controlled, crossover 2) Not controlled, open-label, no crossover, not placebo-controlled 3) Open-label, not controlled, no crossover, not placebo-controlled	Randomized, double-blind, placebo-controlled, crossover	Partially blinded, not controlled, no crossover, not randomized	Not controlled, not blinded, no crossover, not placebo-controlled
Follow-up	6 months	1) 19 months 2) 11 months 3) 3 weeks	?	8 weeks	3 months
Efficacy	No significant improvement	Partially significant improvement, few adverse drug reactions	No significant effect, strong adverse drug reactions	Significant improvement regarding some endpoints	Partially effective, reduction in efficacy, severe adverse drug reactions

### Botulinum toxin

The study conducted by Bertram et al. [7] was a double-blind, placebo-controlled, randomized cross-over study compared the effects of injecting 200 mU of botulinum toxin A with an equal volume 0.9% aline solution into the anterior tibial muscle. Eight female patients with electrophysiologically proven primary orthostatic tremor were randomly assigned to either the experimental or control group, with a drop-out of one patient during the entire course of the study. Following a 20-week period, the patients were switched between the experimental and control groups. The primary outcomes measured included the duration taken to feel unsteady after standing up and the symptom severity which was measured using a visual analogue scale from 0 to a maximum of 10 points. Patients recorded their symptoms twice daily for two weeks in a symptom diary, from which mean values were calculated. In addition, quality of life was assessed using a modified version of the PDQ-39 questionnaire. The study period lasted a total of six months, during which electromyogram analyses were repeated several times. No significant differences were observed in the time required to experience unsteadiness, nor in the symptom diary or the frequency of falls. Electrophysiologically, the results were also similar in both groups [7].

### Gabapentin

Three studies could be found on gabapentin. Evidente et al. [8], reported on seven patients who were included in an open-label study, which was not placebo-controlled. The patients received daily doses of gabapentin ranging from 300 to 1800 mg per day. The initial dosage was set to 300 mg per day, with increments of 300 mg every three to five days until a positive therapeutic effect was achieved. The outcome was measured using a subjective scale assessing improvement in symptoms from 0 to 100%. An improvement of 60–80% as achieved in all patients. Furthermore, four patients also improved in both the severity of symptoms and the time required to experience unsteadiness upon standing. Two patients underwent subsequent electrophysiological examinations, which showed no improvement compared to baseline values prior to the study [8].

Rodrigues et al. [9], described the efficacy of gabapentin in a non-blinded, uncontrolled study involving six patients, focusing on postural instability, electromyogram activity, and quality of life, as assessed by using the PDQ-39. Gabapentin therapy was started with three doses of 300 mg per day and the parameters mentioned above were measured after three weeks. Three of the patients improved their postural instability by up to 70%.Neither adverse drug reactions were recorded, nor an improvement in electrophysiological values. Nevertheless, all patients who completed the questionnaire described a subjective improvement of symptoms.

One double-blind placebo-controlled crossover study by Rodrigues et al. [10] investigated the effect of gabapentin compared to placebo over a two-week period in six patients. In the study, first an open-label administration of ascending doses of gabapentin was performed in all patients to establish the individual maximal effective doses, ranging from 600 to 2700 mg per day. This was followed by a one-week washout period. Thereafter, three patients each were assigned to treatment with either the maximal effective dose of gabapentin or a placebo. Measurement criteria included postural instability and tremor, as well as the patients’ self-assessment using a modified PDQ-39. All patients reported benefits, which persisted throughout an average follow-up period of 19 months. The functional improvements were statistically significant, while only the aspect of emotional well-being showed a significant improvement in the patients’ assessments [10].

### Levetiracetam

A double-blind placebo-controlled crossover study conducted by Hellriegel et al. [11], investigated the efficacy of levetiracetam in comparison to placebo in twelve patients. At the beginning, the patients received 500 mg levetiracetam twice a day, with the dosage subsequently escalated to a maximum of 3000 mg per day for those whose renal function could tolerate such high doses. The primary endpoint of the study was the time to onset of instability in standing position. In addition, parameters including the patient’s swaying, tremor severity and quality of life assessed by the SF-36 questionnaire were recorded. No significant effect was found regarding any of the recorded endpoints. Furthermore, the adverse drug reactions were found to be minimal [11].

### Levodopa

A study conducted by Wills et al. [12], investigated the effects of levodopa in a cohort of eight patients over an eight-week period. Data were collected in the form of a questionnaire with visual analog scales (0–10) to evaluate the patients’ ability to walk and stand, in conjunction with video recordings of five motor tasks relevant to daily life, including standing upright and walking. Initially, a single dose of 200 mg levodopa 50 mg carbidopa was administered, with the first video taken one-hour post-administration. Over the course of the study, the participants received 50 mg levodopa and 12.5 mg carbidopa three times daily, with doses gradually escalated to a maximum of 200 mg levodopa 50 mg carbidopa three times daily. Three patients discontinued the study due to adverse drug reactions. Notably, a significant improvement was observed in the ability to stand upright in the levodopa treated group. However, no effects were observed in the other endpoints, including tandem walking and normal gait. In addition, an average improvement of three points on the visual analog scale was recorded over a period of eight weeks [12].

### Perampanel

A non-blinded study by Gironell and Marín-Lahoz [13], which used no crossover and no placebo controls, involved a cohort of 20 patients. All patients were treated with perampanel and the dosage was increased from 2 mg per day to 4 mg per day after the initial month of treatment. The patients described their perception of improvement on a scale of − 3 to + 3 at both one and three months. Eight patients discontinued due to adverse drug reactions. The reported adverse effects included dizziness, an increased frequency of falls, greater instability, weight gain, and depression. A rebound phenomenon was observed in six of the eight patients who did not continue participating in the study. This manifested itself in the period of two to six weeks after discontinuation of therapy through exacerbation of symptoms compared to the status prior to treatment. Among the remaining patients, 11 of 12 described an improvement. However, this effect diminished and was not observed at the three-month follow-up. Of the 11 patients that had reported an improvement in symptoms at the follow-up at one month, only one patient continued to report such improvement at the three-month follow-up. Eight patients decided to continue treatment with perampanel following the completion of the study [13].

## Discussion

The systematic review of evidence for the treatment of orthostatic tremor revealed a lack of high-quality studies, specifically prospective, randomized, double-blind, placebo-controlled trials. Notably, only one study of this kind was conducted comparing gabapentin to placebo, but with a small number of participants. This study showed an improvement after treatment with gabapentin in comparison to placebo [10]. Therefore, the available evidence for the various treatment modalities is deemed to be of low quality. In addition, the variables used to measure therapeutic efficacy differ considerably across the individual studies. Moreover, no established consensus exists for uniformly assessing orthostatic tremor, which complicates the comparability between different studies. One potential solution to address this issue could be the use of a standardized scale for evaluating orthostatic tremor, such as the OT-10 scale for severity and disability assessment [14].

According to the study by Bertram et al. [7], botulinum toxin was ineffective in treating orthostatic tremor, as demonstrated by the lack of improvement following the administration of 200mU of abobotulinumtoxin A into the anterior tibial muscles. However, no additional studies matching the inclusion criteria were found that investigate the use of botulinum toxin in primary orthostatic tremor. Further studies would be necessary to evaluate the efficacy of higher doses or alternative injection sites. Nevertheless, the potential adverse effects and the risk of destabilization resulting from injections into different muscle groups must be critically assessed. Given the etiology of the tremor to be central, a causal therapeutic approach with botulinum toxin seems not feasible.

With three relevant studies that investigated gabapentin, it is the treatment option with the most substantial evidence for orthostatic tremor [8–10]. Furthermore, these studies also consistently demonstrate a positive effect of gabapentin. However, it should be noted that the sample sizes of each study were very low, resulting in a limited generalizability. Therefore, future studies should involve larger patient cohorts, as well as double-blind randomization and placebo-control. Given that gabapentin is currently regarded as the pharmacological treatment option of choice for primary orthostatic tremor, it would also be necessary to investigate the efficacy of alternative therapies in comparison to gabapentin and/or placebo.

In the investigation conducted by Hellriegel et al. [11], the use of levetiracetam was found to be ineffective in improving the symptoms of orthostatic tremor. It is important to emphasize that high doses up to 3000 mg per day of levetiracetam were used, suggesting that this lack of efficacy was not caused by insufficient dosing. However, no severe adverse effects were reported in any of the patients.

There is one study conducted by Wills et al. [12], investigating the use of levodopa with eight participants, of whom only five completed the study. This study was published alongside a case report. Some individual endpoints exhibited significant improvements with levodopa. The dropout rate of about 40% i noteworthy. It would be necessary to repeat the study with a larger sample size to obtain more substantial evidence of efficacy. It is also currently unclear to what extent the mechanism of action of levodopa can be helpful in this clinical picture, as a dopamine deficiency has not yet been demonstrated [4, 5, 15]. Some authors also emphasize the importance of monitoring for potential tolerance development [12]. Despite the partially positive results, a formal recommendation for the use of levodopa cannot be made currently, as the study in question involved a very small sample size, which was only partially blinded, lacked control, was not randomized and did not incorporate a crossover design, thereby limiting the ability to draw a meaningful comparison. Notably, in a study by Thomas et al. [16], four cases of parkinsonism were described, each marked by the dramatic onset of debilitating standing tremor, which corresponded to the clinical characteristics of orthostatic tremor. In all four cases, the response to levodopa treatment was striking, leading to the complete resolution of the standing tremor. However, this study did not investigate primary orthostatic tremor and only described four cases and therefore did not meet our inclusion criteria [16].

In the study conducted by Gironell and Marín-Lahoz, 92% of the participants showed symptomatic improvement with perampanel [13]. However, a notable amount of adverse drug reactions was documented, which led to a dropout rate of 40% among the initial cohort. As 17 of the 20 participants were also taking other medications for primary orthostatic tremor, the authors suggest that interactions among these medications may have contributed to the severe adverse effects reported. In addition, a reduction or loss of efficacy was observed over the three-month duration of the study. This pronounced development of tolerance was not previously shown in patients taking perampanel due to epilepsy. Given that the tremor study was neither blinded nor randomized and did not include a crossover design, the conclusions regarding clinical applicability are limited. To achieve more generalizable results, controlled studies with larger cohorts would be necessary.

The seizure-suppressive drug primidone, which is mentioned in textbooks as a treatment option, was not included in any of the studies that met the aforementioned criteria [1]. According to the current German S2K guideline [17], the drug is no longer recommended for use in primary orthostatic tremor. The same applies to pramipexole, which is similarly not recommended in the current guideline, despite reports of positive effects in individual patients documented in case reports [17, 18]. Controlled, prospective clinical studies would be necessary to substantiate these effects. No studies were identified regarding clonazepam meeting the above specified criteria, although it is recommended in the German S2k guideline for use in selected cases [17]. Retrospective data, however, indicate a positive effect, underscoring the need for prospective clinical trials in this context [2, 19, 20]. The existence of numerous studies yielding non-significant results raises the question of whether the rarity of the disease and the naturally resulting limitations of sample sizes would negatively impact the validity of statistical tests. This issue can only be resolved through larger multicenter studies. However, it is essential to ensure that diagnostic criteria are clearly defined in order to strengthen the reliability and validity of any study.

None of the studies found significant electrophysiological differences in patients undergoing therapy compared to their pre-therapy state. However, in a case report using perampanel also electrophysiological improvements were detected [21]. In several instances, the electrophysiological differences were deemed insignificant [7, 8, 10, 11], while in other cases, such measurements were not recorded [10, 12, 13]. However, this is an important criterion and should be examined more closely in future studies, as an improvement in electrophysiological outcomes may indicate a restoration of physiological conditions under therapy rather than a mere reduction in symptoms. Further, such findings could provide valuable insights into the etiology of the condition.

One limitation in the comparison of these studies lies in the inconsistent diagnostic criteria employed. In certain studies, this lack of clarity is particularly pronounced. For instance, in the study by Gironell and Marín-Lahoz, it is merely stated that the diagnosis was established by external neurologists [13]. This inconsistency adversely affects comparability and validity. The impact of comorbidities on both therapy and the disease itself must also be critically considered, especially when patients with conditions such as essential tremor are included, as noted in the work by Evidente et al. [8]. Additionally, the studies lack a common primary endpoint, which makes direct comparability difficult. While the potentially prolonged course of the disease is already known, the maximum follow-up reported in one study is only 19 months [20, 22]. Given the expectation of a much longer disease duration, it is prudent to explore extended follow-up periods to identify resistance developments and loss of efficacy. In addition, literature describes that symptoms may intensify over time, raising the question of whether dosage adjustments and/or changes in medication would be necessary in the long-term management to achieve or maintain symptom reduction [22].

## Conclusion

Based on this analysis of the existing literature, we can conclude that gabapentin is the treatment with the strongest evidence for the management of orthostatic tremor. With less supporting evidence, the use of perampanel and levodopa may be recommended as illustrated in Fig. 2. However, it is important to bear in mind that the majority of studies published to date are based on small sample sizes, highlighting the need for larger clinical studies.
